# Which Is More Accurate: Transcutaneous Bilirubin Measurement on the Forehead or Sternum?

**DOI:** 10.34172/aim.2022.88

**Published:** 2022-08-01

**Authors:** Maryam Khoshnoud Shariati, Naeeme Taslimi Taleghani, Neda Izadi, Aghil Miri, Roya Taheri Tafti, Fatemeh Abdollah Gorji

**Affiliations:** ^1^Clinical Research Development Center, Mahdiyeh Educational Hospital, Shahid Beheshti University of Medical Sciences, Tehran, Iran; ^2^Neonatal Health Research Center, Research Institute for Children’s Health, Shahid Beheshti University of Medical Sciences, Tehran, Iran; ^3^Department of Epidemiology, School of Public Health and Safety, Shahid Beheshti University of Medical Sciences, Tehran, Iran; ^4^Clinical Research Development Centers, Mofid Children’s Hospital, Shahid Beheshti University of Medical Sciences, Tehran, Iran; ^5^Shariati Hospital, Tehran University of Medical Sciences, Tehran, Iran

**Keywords:** Neonatal hyperbilirubinemia, Phototherapy, Transcutaneous bilirubin

## Abstract

**Background:**

The accuracy and reliability of noninvasive methods of neonatal jaundice assessment are not completely obvious, including which area of the body is more suitable to estimate actual bilirubin with transcutaneous bilirubinometry (TCB).

**Methods:**

This cross-sectional study compares the accuracy of three noninvasive methods for neonatal jaundice estimation included visual estimation, TCB on the forehead, and TCB on the sternum. The mean and standard deviation describe quantitative variables. In addition to analytical analysis, we used the linear regression test to evaluate the association of different variables with the accuracy of TCB as well as paired *t* test for comparing the TCB results on the sternum with the forehead before and after phototherapy. For all statistical tests, a *P* value less than 0.05 was considered as significant.

**Results:**

We enrolled 100 neonates with a mean age (±SD, standard deviation) of 6.5±1.9 days (range 2–11 days) in our study. The mean gestational age (GA) of the participants was 38.94 weeks±1.00 w SD, and their mean (±SD) weight was 3302 g (±315.60). The mean (mg/dL)±SD for bilirubin level by clinical estimation of jaundice, TCB on the forehead and TCB on the sternum were 17.35±2.88, 17.23±1.63, and 17.77±1.58, respectively. Also, comparing mean differences before and after phototherapy showed that TCB on the sternum is a good predictor for neonatal jaundice before phototherapy (0.539 vs. 0.348).

**Conclusion:**

TCB on the sternum is more predictive than the forehead, especially before phototherapy, to assess the need for treatment in outpatient settings.

## Introduction

 Nearly 60%–85% of all term neonates experience clinical jaundice in the first week of age.^1-3^ As punctures for the consecutive total serum bilirubin (TSB) test are painful and expensive, some parents do not accept monitoring of their neonates’ bilirubin levels. The transcutaneous bilirubin monitoring devices have acceptable reliability compared to visual estimation by measuring the skin color and converting this reading to a bilirubin assessment without pain. Bilirubinometry devices have been used for more than 40 years, and technical improvements have been made to increase their accuracy.^4^ The accuracy of the bilirubin meter is about ± 2 mg/dL according to the results of several studies.^3,4^ For a visual screening of neonatal icterus, Kramer’s rule regarding the cephalocaudal progression of jaundice is accepted.^5,6^ Studies show that the transcutaneous bilirubinometry (TCB) is preferable to clinical evaluation using Kramer’s law.^7^ Daily assessment of jaundice by TCB provides opportunities for predicting the presence of dangerous hyperbilirubinemia in the coming days by providing the actual trajectory plotted against bilirubin monogram. This is mainly practical for infants who are discharged early after birth (before 72 hours). Although the accuracy and reliability of noninvasive techniques for jaundice screening are still reported to be varying,^6^ the AAP recommends routine use of TCB for neonates > 35 weeks to predict the need for phototherapy before hospital discharge.^8^ In addition, it should be noted that the association between serum bilirubin and TCB changes under the influence of phototherapy.^6^ In this study, our goal was to determine which area (on the forehead or sternum) has the best accuracy for performing TCB as a predictor of neonatal jaundice compared to serum bilirubin. It seems that differences in gestational and chronological age or sex of the newborn may affect this issue, requiring extensive demographic studies.

## Materials and Methods

 This cross-sectional descriptive study was performed on icteric term newborns. Our exclusion criteria were prematurity (gestational age ˂ 37 weeks), birth weight ˂ 2500 g, presence of an illness (sepsis, meningitis) or direct hyperbilirubinemia, and history of receiving phototherapy or exchange transfusion. One neonatologist assessed the infant’s eligibility for the study and obtained a signed consent from parents. One expert neonatologist with an experienced nurse (without previous awareness of TCB or TSB) assessed neonates before starting phototherapy. They applied firm pressure to blanch the skin to visualize the color of the underlying subcutaneous tissue. Since proper lighting has a crucial role for discovering subtle jaundice by the eye, we examined newborns under white light. The clinical estimation of the severity of jaundice was done using Kramer’s scale. Based on this scale, five specific ‘dermal zones’ are considered to approximate estimation of jaundice: (1) head and neck: 4–8 mg/dL, (2) upper trunk: 5–12 mg/dL, (3) lower trunk and thighs: 8–16 mg/dL, (4) arms and lower legs: 11–18 mg/d, (5) palms and soles: > 15 mg/dL.^5^ Ordinarily, we applied TCB on the forehead and sternum of all infants before starting phototherapy. We used the KJ-8000 transcutaneous bilirubin meter to check TCB. In order to increase the accuracy of the bilirubin meter, we checked TCB three times consecutively in each area of the skin (the forehead or sternum), and the average was recorded. Also, to determine TSB, blood samples were taken from the neonates before starting phototherapy. The areas of the skin used to check for TCB in the forehead and sternum were hidden from light during phototherapy using a cover. At the end of phototherapy, bilirubin was checked simultaneously in a blood sample from the infant and by TCB on the forehead and sternum. Quantitative variables were described by mean and standard deviation. In analytical analysis, we used the linear regression test to evaluate the associations of different variables with the accuracy of TCB and paired *t* test for comparing the sternal and the forehead TCB results before and after phototherapy. Homogeneity of variables and normality of residuals after linear regression analysis, as well as the assumption underlying paired *t* test and normality of differences were investigated. For all statistical tests, a *P* value less than 0.05 was considered as significant.

## Results

 We enrolled 100 neonates with a mean age ( ± SD, standard deviation) of 6.5 ± 1.9 days (range 2–11 days) in our study, consisting of 50 males (50%) and 50 females (50%). The mean gestational age (GA) of the participants in our study was 38.94 ± 1.00 weeks, and their mean weight was 3302 ± 315.60 g (Table 1). The mean TSB as the standard method for assessing neonatal jaundice before starting phototherapy was 18.68 ± 1.73 mg/dL. The mean (mg/dL) ± SD values of bilirubin level by clinical estimation of jaundice, TCB on the forehead and TCB on the sternum were respectively 17.35 ± 2.88, 17.23 ± 1.63, and 17.77 ± 1.58. The mean difference between TSB before starting phototherapy with TCB on the sternum [-0.91, 95% CI: -1.02 – (-0.79)] was lower than the mean difference between TSB with TCB on the forehead (-1.44, 95% CI: -1.55 – -1.34) and also the mean difference between TSB with clinical estimation [-1.33, 95% CI: -1.87 – -0.79), although all of them were significant (*P* value < 0.001) (Tables 2 and 3). However, the mean difference between clinical estimation with TCB methods on the forehead (0.114, 95% CI: -0.43 – 0.66) and sternum (-0.425, 95% CI: -0.97 – 0.12) were insignificant (*P* value = 0.68 and 0.12, respectively) (Table 2). Our study results showed that not only the mean difference between TCB on the sternum and TSB was significantly lower than the mean difference between TCB on forehead and TSB before phototherapy (0.91 vs. 1.44), but also it was significant after phototherapy (0.82 vs. 1.16) (Table 4). Also, comparing the mean differences before and after phototherapy showed that TCB on the sternum was a good predictor for neonatal jaundice before phototherapy (0.539, 95% CI: 0.464 – 0.613, vs. 0.348, 95% CI: 0.243–0.452) (Table 4). To evaluate the associations of GA, sex, and age of the neonates with the accuracy of TCB, the linear regression test was used. None of these factors affected TCB before phototherapy (Table 5).

**Table 1:** Demographic Characteristics of Neonates Included in the Study for Hyperbilirubinemia by Three Methods: Clinical, Transcutaneous, Serum Measurement

**Variable**		**Mean**	**SD**	**Range**	
				**Min**	**Max**
Girl (n = 50)	GA (wk)	38.8	1.03	37.6	42.1
	BW (g)	3295	348.76	2830	4150
	Age (d)	6.72	1.85	4	11
Boy (n = 50)	GA (wk)	39.0	0.96	38	42
	BW (g)	3309.6	281.96	2900	4050
	AGE (d)	6.22	1.89	2	10
Total N = 100	GA (wk)	38.94	1.00	37.6	42.1
	BW (g)	3302	315.60	2830	4150
	Age (d)	6.47	1.88	2	11

BW, Birth Weight; GA, gestational age; SD, standard deviation.

**Table 2:** Comparison of Clinically Estimated Bilirubin Level with TCB on the Forehead, TCB on the Sternum and Serum Bilirubin Level before Phototherapy

**Variables**	**Descriptive**				**Paired ** ***t*** **-test**			
	**Mean**	**SD**	**95% Confidence Interval**		**Mean Diff**	**95% Confidence Interval of Difference**		***P *** **Value**
			**Lower**	**Upper**		**Lower**	**Upper**	
Clinical evaluation	17.35	2.88	16.77	17.92	-1.33	-1.87	-0.79	< 0.0001
TSB	18.68	1.73	18.34	19.02				
Clinical evaluation	17.35	2.88	16.77	17.92	0.114	-0.43	0.66	0.68
TCB (forehead)	17.23	1.63	16.91	17.56				
Clinical evaluation	17.35	2.88	16.77	17.92	-0.425	-0.97	0.12	0.12
TCB (sternum)	17.77	1.58	17.46	18.08				

SD, standard deviation; TCB, transcutaneous bilirubinometry.

**Table 3:** Comparison of Serum Bilirubin Level with TCB on the Forehead and TCB on the Sternum before Phototherapy

**Variables**	**Descriptive Statistics**				**Paired ** ***t*** **-test Statistics**			
	**Mean**	**SD**	**95% Confidence Interval**		**Mean Diff**	**95% Confidence Interval of Difference**		***P *** **Value**
			**Lower**	**Upper**		**Lower**	**Upper**	
TSB	18.68	1.73	18.34	19.02	-1.44	-1.55	-1.34	< 0.0001
TCB (forehead)	17.23	1.63	16.91	17.56				
TSB	18.68	1.73	18.34	19.02	-0.91	-1.02	-0.79	< 0.0001
TCB (sternum)	17.77	1.58	17.46	18.08				

SD, standard deviation; TCB, transcutaneous bilirubinometry.

**Table 4:** Comparison of TCB on the Sternum with TCB on the Forehead (before and after Phototherapy)

		**Descriptive Statistics**				**Paired ** ***t*** ** test Statistics**			
		**Mean**	**SD**	**95% Confidence Interval**		**Mean Diff**	**95% Confidence Interval of Difference**		***P*** ** Value**
				**Lower**	**Upper**		**Lower**	**Upper**	
Before phototherapy	Difference between forehead TCB and TSB	1.449	0.54	1.34	1.55	0.539	0.464	0.613	< 0.0001
	Difference between sternum TCB and TSB	0.91	0.56	0.79	1.02				
After phototherapy	Difference between forehead TCB and TSB	1.16	0.48	1.07	1.26	0.348	0.243	0.452	< 0.0001
	Difference between sternum TCB and TSB	0.82	0.53	0.71	0.92				

TSB, total serum bilirubin; TCB, transcutaneous bilirubinometry.

**Table 5:** Association of Gestational Age and Newborns’ Sex and Age with the Accuracy of TCB before Starting Phototherapy Using Linear Regression

**Variables**	**TCB (forehead)**					**TCB (sternum)**				
	**Coefficient**	**SE**	**95% Confidence Interval of Difference**		***P*** ** Value**	**Coefficient**	**SE**	**95% Confidence Interval of Difference**		***P*** ** Value**
			**Lower**	**Upper**				**lower**	**Upper**	
Age (days)	0.099	0.029	-0.048	0.067	0.73	0.020	0.030	-0.039	0.081	0.49
Sex (boy)	-0.217	0.109	-0.434	-0.0002	0.05	-0.004	0.114	-0.230	0.222	0.97
Gestational age (wk)	0.074	0.054	-0.033	0.182	0.17	0.060	0.057	-0.052	0.174	0.28

SE, standard error; TCB, transcutaneous bilirubinometry.

## Discussion

 In the present study, we compared the visual estimation of jaundice and TCB on the forehead and sternum with the standard TSB test. Our main goal was evaluating the true estimation power of TCB with the examination of its quantity differences with the TSB and finding factors that affect TCB accuracy. Numerous studies have been performed to evaluate the various aspects of the accuracy of TCB by examining the similarity of their results with TSB. Since factors such as skin color, GA, race, history of phototherapy, and severity of jaundice affect the TCB results, there is controversy about its acceptability rate.^3^ Therefore, with the improvement of human knowledge in technologies for the production of bilirubinometer, new perspectives are still emerging for convenient diagnosis of neonatal jaundice.

 In the present study, no significant difference was found between the clinical estimation of jaundice by examiner vision and the TCB method. In other words, if the visual estimate of jaundice is done in adequate lighting by a skilled person, it can be as efficient as bilirubinometers. The importance of clinical assessment of infantile jaundice by observation as the first step at home obvious, but can this method be trusted? In the present study, the mean difference between the clinical estimation of neonatal jaundice by an experienced nurse and neonatologist was significant compared to TSB. However, it should be remembered that only term infants weighing more than 2500 g and aged 2–11 days were included in the present study. A study conducted in Israel by RISKIN in 2008 on 1129 full-term and near-term neonates with a mean age of 7 days reported a mean serum jaundice of 6.7 ± 2.9 mg/dL and a mean visual jaundice estimate of 6.6 ± 3.2 (Pearson’s r = 0.752, *P* < 0.0001). The results of this study showed that although there was a good correlation between BiliEye (visual jaundice estimation) and the actual level of TSB, visual assessment as a screening tool for the diagnosis of significant neonatal hyperbilirubinemia before discharge was unreliable. Infants with TSB levels in high-risk areas may be clinically diagnosed as low-risk and therefore, may not be adequately followed up.^7^ In a cross-sectional study in India, Alok Yadav (published in 2020) evaluated the ability of 505 mothers to diagnose the jaundice of their neonates with GA over 34 weeks. They considered the appearance of visible jaundice up to the leg area as significant jaundice, and subsequently, checked these infants for TSB. The sensitivity, specificity, positive predictive value (PPV), and negative predictive value (NPV) of detection of significant neonatal jaundice by mothers were 51.47%, 88.33%, 39.29%, and 92.12%, respectively, with 83.3% accuracy (95% CI–9.75%–86.46%). While the sensitivity, specificity, PPV and NPV detection of significant jaundice by pediatricians were 85.29%, 85.35%, 47.54%, 97.39%, respectively with 85.35% accuracy (95% CI–81.96%–88.32%). Although these results showed good specificity and acceptable NPV, the sensitivity of maternal ability for diagnosing very low or high jaundice was poor.^8^ Shafiq et al in 2017 studied 249 infants aged 2–7 days and GA more than 35 weeks at Shafa Hospital in Pakistan in a cross-sectional study. The aim was to determine the validity of the absence of visually detectable jaundice under the nipples to rule out significant jaundice. The results showed that lack of significant jaundice below the nipple has a sensitivity of 100% and specificity of 73.23%.^9^

 Keren et al designed a prospective cohort study to appraise the clinical assessment ability to predict the presence of high jaundice before discharge. In this study, 522 term and near-term infants were evaluated for jaundice by nurses. Evaluation of jaundice by nurses had only a moderate correlation with bilirubin concentration and was similar in black and non-black infants (Spearman’s rho = 0.45 and 0.55, respectively; *P* = 0.13). Correlation was particularly weak in neonates at ≤ 38 weeks gestation (rho = 0.29) compared with neonates > 38 weeks gestation (rho = 0.53, *P* = 0.05). Their results showed that the rate of cephalocaudal progression of jaundice could not accurately estimate bilirubin levels, especially in late preterm infants. However, the absence of complete clinical jaundice can predict with great accuracy that high serum bilirubin will not be developed.^10^ Therefore, considering the GA of neonates and the small sample size of our study, our suggestion is the use of visual assessment of jaundice only by an experienced person for term neonates and in low-income countries as a substitute for TCB.

 In the present study, the mean difference between TSB and TCB on the sternum and forehead was -0.91 (95% CI: -1.02 – -0.79) and -1.44 (95% CI: -1.55 – -1.34) with *P* value ˂ 0.0001, respectively. Consistent with the results of our study, Johnson et al, in a prospective study on 35 neonates over 35 weeks, showed that TCB can lead to overtreatment at low bilirubin levels while it is not accurate enough for high bilirubin levels. The mean difference between TSB and TCB in that study was 8.4 (-18.5 – 35.3).^11^ Contrary to the results of our study, a retrospective cross-sectional study by Ying in 2020 on neonates over 35 weeks and 2 kg showed a strong association between TSB and TCB with a correlation coefficient of 0.708 (*P* < .001), but this association was not very satisfying in premature infants with high bilirubin levels.^12^

 On the other hand, considering the mean difference between TSB and TCB on the forehead and sternum in our study (Table 4), the result shows that sternal TCB, especially before phototherapy, is closer to the TSB. Accordingly, it can be concluded that the accuracy of sternal TCB compared is higher to the forehead, especially before starting phototherapy, because this area is covered with clothes.

 A few studies have assessed which areas of the body yield TCB findings closer to serum bilirubin levels.^12,13^ The results of several studies show superiority of the forehead and sternum compared to the back and thighs.^14,15^ Consistent with the results of our study, in El-Kabbany and colleagues’ study on 316 infants with GA between 32 to 40 weeks and aged 1 to 15 days, the difference (bias) between TSB with forehead TCB and sternal TCB before phototherapy was -3.1 to 4 mg/dL with a mean bias of 1.1 ± 1.1, and -3 to 3.4 with a mean bias of 1.1 ± 0.4, respectively. They showed not only a statistically significant difference between the mean values of TSB and TCB, but the mean difference between TSB and sternal TCB was less than the mean difference between TSB and forehead TCB.^2^

 The correlation coefficients between the TSB and TCB on the forehead and sternum were reported at 0.914 and 0.953, respectively, by Maisels. In other words, this study showed a slightly stronger association between sternal TCB and TSB, especially in outpatients.^15^ However, unlike Maisels’ study, in a recent study by Lucanova et al, the correlation coefficient between the TSB and forehead TCB was reported to be significantly higher than the sternal region.^16^ Although GA, postnatal age, and ABO hemolytic disease are possible causes of the difference between TCB and TSB in some reports,^12,17,18^ it is not confirmed in all studies. For example, Cucuy et al found a good correlation coefficient between TCB and TSB in preterm infants, but they found no association for the baby’s chronological age, other morbidities, history of receiving phototherapy, and the time interval between completing phototherapy and performing a skin test.^19^ Most studies have shown similar results regarding the unreliability of skin tests for high bilirubin levels.^16-18^ In the present study, there was no effect for GA and the infant’s sex and age on the accuracy of TCB before starting phototherapy. The distinguishing factor of the present study is assessing in which area of the body TCB can be more accurate in estimating bilirubin. Since this issue has been studied only in a few trials, the discrepancy in the results raises the need for further studies in the future in order to be included in the guidelines if confirmed. It seems that the small sample size may be one of the limitations of the present study. In addition, in future studies, the factors affecting the accuracy of TCB results should be investigated, such as the time of performing TCB (before or after hospital discharge) and the severity of hyperbilirubinemia.

 In conclusion, TCB on the sternum is more predictive than on the forehead, especially before phototherapy, to assess the need for treatment in outpatient settings.
